# Endoscopic versus surgical treatment for infected necrotizing pancreatitis: a systematic review and meta-analysis of randomized controlled trials

**DOI:** 10.1007/s00464-020-07469-9

**Published:** 2020-02-28

**Authors:** C. M. Haney, K. F. Kowalewski, M. W. Schmidt, R. Koschny, E. A. Felinska, E. Kalkum, P. Probst, M. K. Diener, B. P. Müller-Stich, T. Hackert, F. Nickel

**Affiliations:** 1 Department of General, Visceral and Transplantation Surgery, Heidelberg University Hospital, Im Neuenheimer Feld 110, 69120 Heidelberg, Germany; 2 Department of Gastroenterology, Heidelberg University Hospital, Im Neuenheimer Feld 410, 69120 Heidelberg, Germany; 3 The Study Center of the German Surgical Society (SDGC), University of Heidelberg, Im Neuenheimer Feld 130.3, 69120 Heidelberg, Germany

**Keywords:** Acute pancreatitis, Necrosectomy, Endoscopy, Randomized controlled trials, Systematic review

## Abstract

**Objective:**

To compare outcomes of endoscopic and surgical treatment for infected necrotizing pancreatitis (INP) based on results of randomized controlled trials (RCT).

**Background:**

Treatment of INP has changed in the last two decades with adoption of interventional, endoscopic and minimally invasive surgical procedures for drainage and necrosectomy. However, this relies mostly on observational studies.

**Methods:**

We performed a systematic review following Cochrane and PRISMA guidelines and AMSTAR-2 criteria and searched CENTRAL, Medline and Web of Science. Randomized controlled trails that compared an endoscopic treatment to a surgical treatment for patients with infected walled-off necrosis and included one of the main outcomes were eligible for inclusion. The main outcomes were mortality and new onset multiple organ failure. Prospero registration ID: CRD42019126033

**Results:**

Three RCTs with 190 patients were included. Intention to treat analysis showed no difference in mortality. However, patients in the endoscopic group had statistically significant lower odds of experiencing new onset multiple organ failure (odds ratio (OR) confidence interval [CI] 0.31 [0.10, 0.98]) and were statistically less likely to suffer from perforations of visceral organs or enterocutaneous fistulae (OR [CI] 0.31 [0.10, 0.93]), and pancreatic fistulae (OR [CI] 0.09 [0.03, 0.28]). Patients with endoscopic treatment had a statistically significant lower mean hospital stay (Mean difference [CI] − 7.86 days [− 14.49, − 1.22]). No differences in bleeding requiring intervention, incisional hernia, exocrine or endocrine insufficiency or ICU stay were apparent. Overall certainty of evidence was moderate.

**Conclusion:**

There seem to be possible benefits of endoscopic treatment procedure. Given the heterogenous procedures in the surgical group as well as the low amount of randomized evidence, further studies are needed to evaluate the combination of different approaches and appropriate timepoints for interventions.

**Electronic supplementary material:**

The online version of this article (10.1007/s00464-020-07469-9) contains supplementary material, which is available to authorized users.

Acute pancreatitis is a disease with potentially lethal outcome. While about 80% of patients only suffer of mild pancreatitis, 20% of patients progress to necrotizing pancreatitis [1] These patients are currently treated with a primary conservative approach and interventions are postponed if possible until the necrosis becomes walled off and liquified. While sterile walled-off necrosis only requires intervention for symptoms such as pain, vomiting, early satiety and/or enlarging size, this is not the case in patients with infected necrosis. These patients often require intensive care and long hospital stays and can present mortality rates of 15 to 34% [2–4] Traditionally speaking, infected necrotizing pancreatitis (INP) is seen as an indication for interventional or surgical treatment even though the added inflammatory response after the treatment can further exacerbate the course of the disease with resulting multiple organ failure, bleeding, or injury to organs. While different treatment approaches have been proposed, few randomized controlled trials (RCT) have been performed. In an RCT by van Santvoort et al. a minimally invasive step-up approach with initial drainage and if needed subsequent video-assisted retroperitoneal debridement was shown to be superior to the primary open surgical approach for patients with confirmed or suspected INP. This was the case in both short- and long-term outcome [2, 5]. Therefore, standard treatment for INP has shifted from open surgical treatment to step-up and minimally invasive approaches in recent years [1].

With the evolution of endoscopy in recent years, endoscopic approaches have gained popularity for treatment of INP. Multiple methods have been proposed ranging from step-up procedures in which one or multiple plastic or more recently lumen-apposing metal stents are placed to drain the fluids and then only if necessary the remaining necrosis is removed to direct necrosectomy [6–9]. The rising popularity of this approach relies mostly on reports of observational studies from expert tertiary centers [9–11]. These have shown promising results with relatively low mortality and morbidity [10].

Therefore, it is important to compare the outcomes of these two treatments in systematic reviews. However, most recent systematic reviews comparing endoscopy to surgery for INP included both RCTs as well as observational studies or have methodological limitations and do not provide a judgement of the certainty of the available evidence [10, 12–14].

This systematic review aims to compare the available randomized evidence comparing endoscopic treatment (endoscopic drainage and necrosectomy) to surgical treatment (surgical drainage and necrosectomy) for INP. The main outcomes that of this review are the highly relevant postinterventional mortality and new onset multiple organ failure. By only including RCTs, the commonly present bias of observational studies is avoided.

## Methods

This review follows the Cochrane Handbook of Systematic Reviews and Interventions [15] and is in concordance of the AMSTAR-2 criteria [16] and the PRISMA guidelines [17]. It was prospectively registered on PROSPERO (ID: CRD42019126033).

### Eligibility criteria

Only trials fulfilling the following PICOs criteria were eligible to be included.

P (patients): Patients with confirmed or suspected infected necrotizing pancreatitis eligible for both endoscopy and surgery.

I (intervention): Endoscopy (either step-up or non-step-up procedures).

C (control): Surgery (either step-up or non-step-up procedures).

O (outcome): At least one of the main outcomes (mortality or new onset multiple organ failure).

S (study type): Only randomized controlled trials.

### Information sources

The following databases were searched according to Goossen et al. [18]:

1. Cochrane Central Register of Controlled Trials (CENTRAL)
2. Medline (via Pubmed)
3. Web of Science

The last database search was performed on April 24, 2019. In addition to the databases listed above we conducted a search for ongoing trials (International Clinical Trials Registry Platform, last search May 12, 2019) and a web search for further trials. An additional hand search was performed and content experts in the field of pancreatic surgery (T.H., O.S., M.K.D., B.P.M.S.) were consulted as to whether there were possibly more studies that were not found by the search.

Authors of ongoing studies were contacted for more information and potentially includable data.

### Search

We performed the search with a combination of Medical subject headings (MeSH) and free text words combined by Boolean connectors. The search strategy for Medline was the following:

(((necrot*[tiab] AND (pancreatitis[tiab] OR pancreatic[tiab] OR pancreas[tiab])) OR "Pancreatitis, Acute Necrotizing"[Mesh].

AND

(endoscop*[tiab] OR surgery[tiab] OR surgeries[tiab] OR surgical[tiab] OR operation*[tiab] OR therap*[tiab] OR “minimally invasive”[tiab] OR “endoscopic transgastric approach”[tiab] OR ETA[tiab] OR necrosectom*[tiab] OR transgastric[tiab] OR transluminal[tiab])).

OR "Pancreatitis, Acute Necrotizing/surgery"[Mesh] OR "Pancreatitis, Acute Necrotizing/therapy"[Mesh]).

AND

random*[tiab] OR RCT*[tiab] OR “Randomized Controlled Trial”[pt] OR "Randomized Controlled Trials as Topic"[Mesh] OR "Controlled Clinical Trials as Topic"[Mesh].

### Study selection

Two reviewers (CMH and EAF) performed title and abstract screening independently. After the title and abstract screening, the reviewers assessed the full texts for inclusion and evaluated if the criteria for inclusion were met. The reference lists of the included studies were searched for further studies that might meet the inclusion criteria. Any disagreement at the different stages was resolved either by discussion or a third party (KFK). All included studies were saved in an EndNote database (Version X9, Clarivate Analytics, Philadelphia, United States).

### Data collection process

Data of the included studies were extracted via a predefined piloted extraction sheet by two reviewers (CMH and EAF) independently and then compared. Differences were solved by discussion or by a third party (KFK). The data were pooled in an Excel-sheet.

### Data items

Extracted data were the following: (1) general study information, (2) baseline data of study participants, (3) type of intervention, (4) primary and secondary outcomes, (5) funding.

### Risk of bias in individual studies

Risk of bias was assessed using the Cochrane risk of bias tool 2.0 version of March 15, 2019 [19]. Due to the publishing of an updated risk of bias tool this presents a deviation from the PROSPERO registration. Two raters (CMH and MWS) assessed all studies independently and all outcomes in the five prespecified domains and rated risk of bias with the help of signaling questions which are provided by the risk of bias 2.0 tool. Disagreement was solved by discussion or inclusion of a third rater (KFK). As proposed by Probst et al. [20] different domains of blinding were assessed in order to adequately rate the impact that blinding could have had on the outcomes. Funding was included as a potential risk of bias as it has been shown that industry funding can influence the outcome of studies [21]

### Publication bias

As < 10 studies were included in this meta-analysis, publication bias was not able to be assessed in this review [15].

### Certainty in evidence

We assessed the certainty of evidence using the GRADE approach [4] with the help of the Grade Pro Software (McMaster University and Evidence Prime Inc, Ontario, Canada). A third reviewer (KFK) solved conflicts if the two primary reviewers (CMH and MWS) that worked independently did not find a consensus.

### Summary measures and synthesis of data

Review manager (Revman version 5.3, The Cochrane Collaboration, The Nordic Cochrane Centre, Copenhagen, Denmark) was used to pool and quantitatively summarize the endpoints. The odds ratio (OR) and 95% confidence interval (CI) was calculated using the Mantel–Haenszel model for dichotomous data such as mortality or multiple organ failure. For continuous data such as length of hospital stay the mean difference (MD) with CI was calculated using the inverse variance model. If data were presented in the original paper other than mean and standard deviation, we recalculated this data using the methods described by Hozo et al. [22] and Higgins and Green [15]. Due to the differing treatments and differing populations we used a random effects model for all calculations. Heterogeneity was investigated with the *X*^2^ and *I*^2^ test and interpreted as follows: 0–40% low, 30–60% moderate, 50–90% high and 75–100% considerable [23]. Pooled analyses were visualized with Forest plots. If studies performed a per protocol (PP) or modified intention to treat (mITT) analysis, the data of the missing patients were tried to be retrieved and analyzed separately in an intention to treat (ITT) analysis.

## Results

### Study selection

**Fig. 1:**
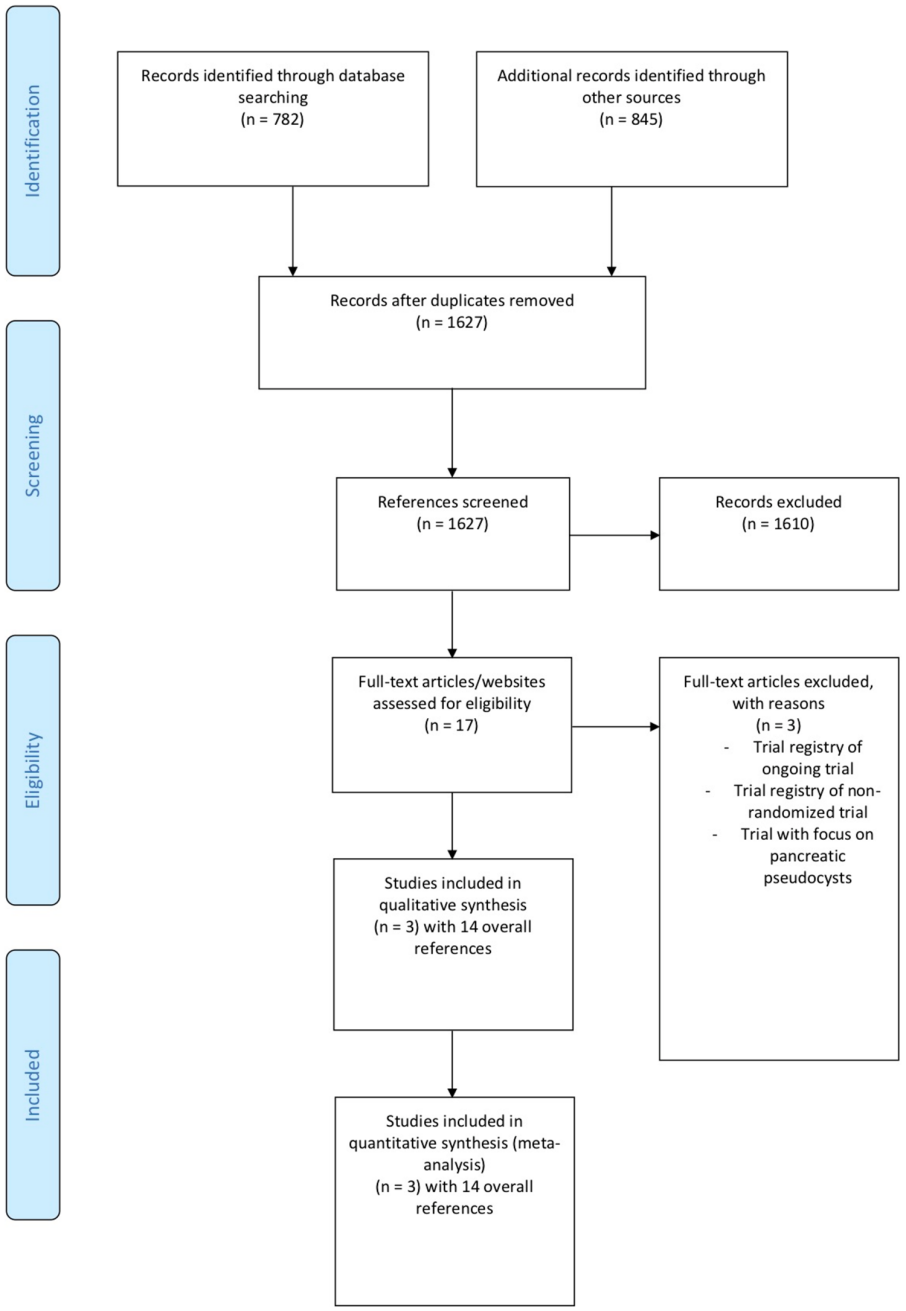
PRISMA flow chart

1627 references were title and abstract screened for inclusion. Of these 1610 were excluded due to ineligibility. 17 references were full text screened and of these 3 were excluded as one reference presented the trial registry of a non-randomized trial and one reference presented the trial registry of an ongoing trial [24], while one randomized trial identified by hand search presented a trial that excluded patients with > 30% necrotic debris in the walled-off necrosis. Furthermore, 18.3% of the patients in the trial were patients with pseudocysts and the patients with walled-off necrosis had sterile walled-off necrosis [25]. The remaining 14 references were all related to the three included studies (trial registry [26–28], congress abstracts or comments [29, 34], protocols [35, 36], final publications [37–39]). The full search process is presented in Fig. 1.

### Study characteristics

The three studies added up to 184 patients that were analyzed in the mITT analysis and 188 patients in the ITT analysis (mITT: range 20–98, ITT: range 22–98) and were conducted in two (Netherlands and United States of America). The PENGUIN-trial included 20 patients in the mITT analysis and was a pilot study with the main outcome being the difference of Il-6 rise after treatment [37]. Both the TENSION-trial and the MISER-trial presented a composite of major complications and death as the main outcome [38, 39]. However, these composites did not entirely follow the same definitions. Inclusion criteria were similar for all trials. For further details on trial design and interventions please see Table 1. Further details of population characteristics are presented in Table 2.

**Table 1:** Trial design and interventions (*RCT* randomized controlled trial, *VARD* video-assisted retroperitoneal debridement, *ITT* intention to treat, *mITT* modified intention to treat)

Study information	PENGUIN-trial	TENSION-trial	MISER-trial
First author	O.J. Bakker	S. van Brunschot	J. Y. Bang
Year published	2012	2018	2019
Country	Netherlands	Netherlands	United States of America
Design	RCT, multicentric	RCT, multicentric	RCT, monocentric
Trial time period	2008–2010	2011–2015	2014–2017
Primary outcome	Postprocedural serum interleukin 6 levels	Composite outcome of death and major complications	Composite outcome of death and major complications
Stage of pancreatitis	Infected necrotizing pancreatitis or suspected infected necrotizing pancreatitis	Infected necrotizing pancreatitis or suspected infected necrotizing pancreatitis	Infected necrotizing pancreatitis or suspected infected necrotizing pancreatitis
Timing of intervention	Intervention was postponed to at least a month after onset of disease whenever possible	Randomization and intervention postponed until 4 weeks after onset of pancreatitis whenever possible	Randomization postponed until better demarcation of necrosis if necessary
Inclusion criteria	Confirmed or suspected infected necrotizing pancreatitis eligible for both endoscopic or surgical necrosectomy	Confirmed or suspected infected necrotizing pancreatitis eligible for both endoscopic or surgical necrosectomy	Confirmed or suspected infected necrotizing pancreatitis eligible for both endoscopic or surgical necrosectomy
Exclusion criteria	- Previous surgical or endoscopic necrosectomy - Previous exploratory laparotomy - Pancreatitis as consequence of abdominal surgery - Flare up of chronic pancreatitis - Abdominal compartment syndrome - Perforation of visceral organ - Bleeding as indication for intervention	- Previous invasive interventions for necrotizing pancreatitis - Chronic pancreatitis - Recurrent acute pancreatitis - Indication for emergency laparotomy	- Prior surgical or endoscopic drainage or necrosectomy - Pancreatitis secondary to trauma or surgical intervention - Presence of indwelling percutaneous catheters before randomization - Chronic pancreatitis - Pregnancy
Patients randomized	22	98	70
Patients analyzed by trial authors	20	98	66
Method of analyzing	mITT	ITT	mITT
Excluded patients	2 (patients excluded due to clinical improvement after drainage)	0	4 (2 Patients excluded due to clinical improvement after drainage, 2 patients excluded due to protocol violation)
Data of excluded patients	Confirmation acquired from authors that no further complications or mortality occurred in patients excluded due to clinical improvement	No further data required	Patients excluded due to “resolution of symptoms after percutaneous drainage” included in ITT analysis under the assumption that they showed no further complications. Patients excluded due to protocol violations were not included in analysis
Endoscopic treatment	Endoscopic drainage and subsequent endoscopic necrosectomy	Endoscopic drainage and if necessary subsequent endoscopic necrosectomy. 2, 7 Fr plastic stents used	Endoscopic drainage and if necessary subsequent endoscopic necrosectomy. 2, 7 Fr plastic stents or metal stents or more than one drainage sites (multi-gateway)
Surgical treatment	- VARD following previously placed retroperitoneal percutaneous drain OR - Open necrosectomy if VARD not possible	- Percutaneous drainage and if necessary subsequent VARD	- Laparoscopic cystogastrostomy with pancreatic necrosectomy OR - Percutaneous drainage and subsequent VARD
Quality control	Expert panel of gastrointestinal surgeons, gastroenterologists and radiologists evaluated candidates prior to randomization	Expert panel of gastrointestinal surgeons, gastrointestinal endoscopists and radiologists evaluated candidates prior to randomization	Expert panel of gastrointestinal surgeons, gastroenterologists, and radiologists evaluated candidates prior to randomization
Funding of study	First author received grant from Netherlands Organization for Health Research and Development to perform clinical trials	Dutch Digestive Disease Foundation, Fonds NutsOhra, and the Netherlands Organization for Health Research and Development	No mention of special trial funding

	Endoscopy (10)	Surgery (12)	Endoscopy (51)	Surgery (47)	Endoscopy (34)	Surgery (34)
Only drainage	0	2	22	24	22	2
Step-up procedure (initial drainage and subsequent necrosectomy)	9 (one patient received immediate necrosectomy after drainage)	8	26	22	11 (one patient received immediate necrosectomy after drainage)	9
Direct necrosectomy without previous drainage	0	2	0		0	23
Spontaneous improvement without treatment	0	0	1	1	0	0
Further/differing treatment OR subsequent conversion to other treatment modality	6 (2 patients received additional VARD after multiple (5/7) unsuccessful endoscopic necrosectomies, 4 patients received percutaneous catheter drainage before endoscopic treatment)		16 (2 to surgical step-up after unsuccessful endoscopy, 14 patients received additional percutaneous drainage)	2 (2 patients received additional endoscopic drainage)	20 (6 patients received additional percutaneous drainage after initial endoscopic drainage, 14 patients had an indwelling percutaneous catheter at time of intervention)	6 (One patient underwent open necrosectomy, 2 patients underwent endoscopic necrosectomy after aborted surgical necrosectomy; 3 patients underwent endoscopic necrosectomy (1) or drainage (2) after no clinical improvement after surgery)

**Table 2:** Population baseline characteristics as described by the authors of trials (data are mean (SD) or median (IQR); *APACHE* Acute Physiologic Assessment and Chronic Health Evaluation, *ICU* intensive care unit, *ASA* American Society of Anesthesiologists; ^a^ASA status at admission, ^b^ASA status at intervention)

Patient characteristics	PENGUIN-trial		TENSION-trial		MISER-trial							
	Endoscopy (10)	Surgery (10)	Endoscopy (51)	Surgery (47)	Endoscopy (34)				Surgery (32)			
Age in years	62 (58–70)	64 (46–72)	63 (14)	60 (11)	55.6 (14.2)				52.9 (14.2)			
Men (%)	60	80	67	62	65				66			
BMI (mean)	29 (26–35)	27 (23–37)	29 (25–32)	28 (25–30)	Not available				Not available			
Cause of pancreatitis												
Biliary cause of pancreatitis (%)	60	70	51	64	41				25			
Alcohol as cause for pancreatitis (%)	20	20	14	15	18				34			
Other cause of pancreatitis (%)	20	10	35	21	41				41			
Severity of illness												
CT-severity score	8 (4–10)	8 (4–10)	6 (6–8)	8 (6–10)	4–6		8–10		4–6		8–10	
					3%		97%		9%		90%	
APACHE II score	10 (6–14)	11 (7–14)	9 (5–13)	10 (6–13)	30 (26–35)				21 (16–23)			
Time since onset until intervention (days)	48 (36–74)	59 (29–69)	39 (28–54)	41 (28–52)	< 28	28–42		> 42	< 28	28–42		> 42
					27%	56%		18%	22%	50%		28%
Infected necrosis (%)	100	90	90	98	91				94			
Admitted to ICU at randomization (%)	20	30	41	53	71				66			
Multiple organ failure 24 h prior to randomization (%)	20	10	18	15	21				22			
SIRS prior to randomization (%)	90	70	65	81	47				50			
Single organ failure prior to randomization (%)	20	30	25	30	6				9			
ASA 1 status (%)	10^a^	10^a^	33^a^	38^a^	0^b^				0^b^			
ASA 2 status (%)	90^a^	80^a^	57^a^	57^a^	6^b^				3^b^			
ASA 3 status (%)	0^a^	10^a^	10^a^	4^a^	76^b^				72^b^			
ASA 4 status (%)	0^a^	0^a^	0^a^	0^a^	18^b^				25^b^			

### Risk of bias within studies

**Fig. 2:**
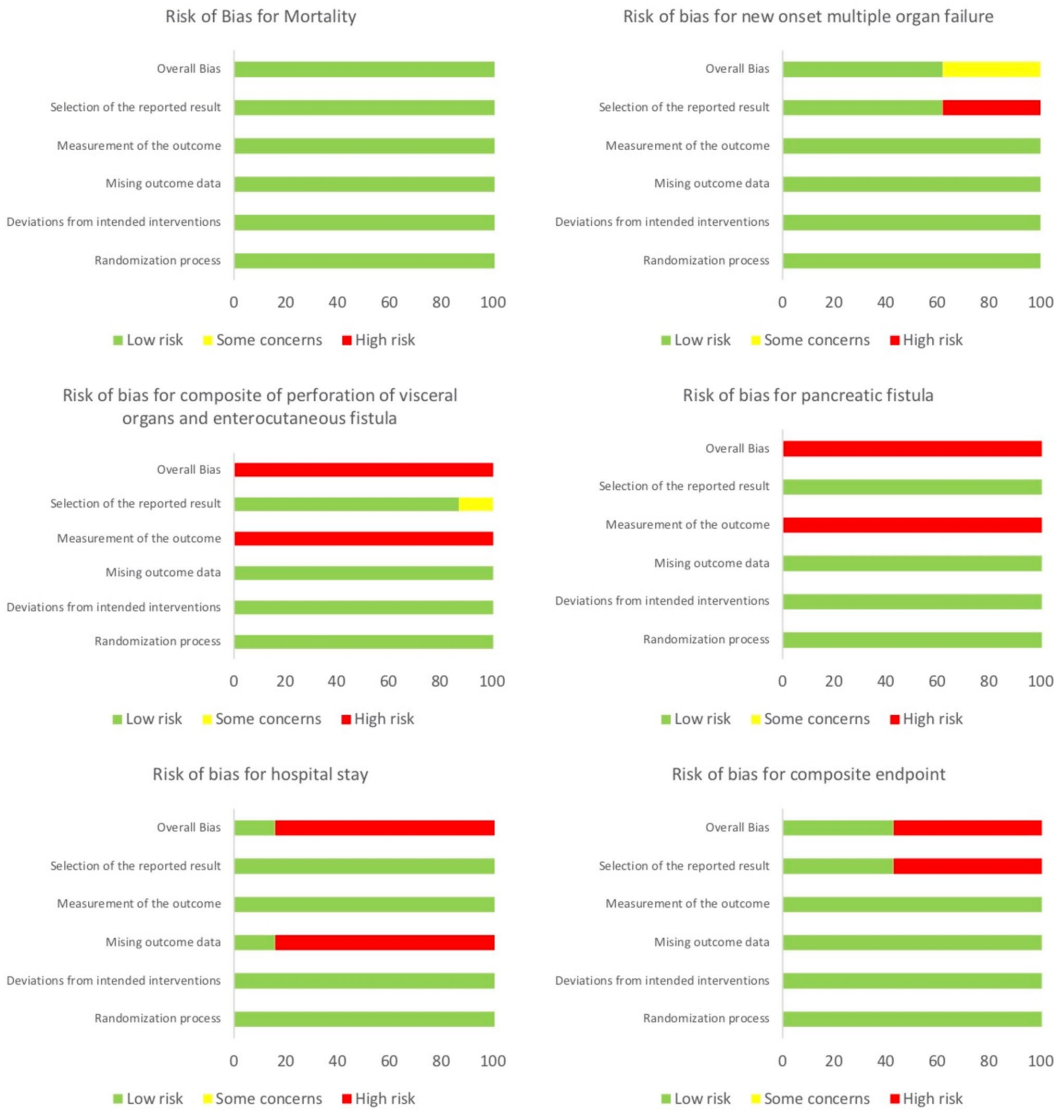
Risk of Bias for most important outcomes, risk of bias assessed with the Cochrane Risk of Bias Tool 2.0, Risk of Bias for further outcomes presented in the Supplementary material

Risk of bias was assessed for all outcomes. The risk for the most important outcomes is presented in Fig. 2. Risk of bias for further outcomes is presented in the supplementary material. Overall, there was not a high risk of bias for the main outcomes as they followed prespecified or objective criteria.

Both the outcomes pancreatic fistula and perforation of visceral organ/enterocutaneous fistula were rated as being of high risk of bias in all studies. This was because it was decided that there was a detection bias as both outcomes in part rely on presence of a percutaneous catheter or surgical wound site. As the endoscopic groups are less likely to have percutaneous drainage catheters and surgical wound even if they have a fistula, these are less likely to be detected. The risk of bias for the hospital stay was rated as high as there were data missing on patients from the surgical group that showed clinical improvement after catheter drainage and were likely to have a lower hospital stay than the average surgical patient. Therefore, this could have influenced the overall result. The composite endpoint and main outcome of the MISER-trial was rated to be of high risk of bias as it included pancreatic fistulae, and this is as discussed above an outcome that suffers from a detection bias. The risks of bias were taken into consideration when rating the certainty of evidence. The full GRADE evidence table with the reasons for the grading of the evidence has been provided as a supplementary file.

### Main outcomes

We performed two sets of analysis following these definitions:

mITT: Patients were analyzed as was reported in the study.

ITT: Patients were analyzed as they were randomized i.e., patients excluded in two studies because of resolution of symptoms after drainage were included in the analysis (Endoscopy: *n* = 0; Surgery: *n* = 4). As data on patients excluded due to protocol violations were not available, these patients were not included (Endoscopy: *n* = 1; Surgery *n* = 1). However, there were no changes of significance due to different forms of analysis. The definitions of all outcomes are presented in the supplementary material.

#### Mortality

Mortality was reported in all trials. In both the mITT (OR [CI] 0.99 [0.32, 3.01]) and the ITT (OR [CI] 1.12 [0.44, 2.85]) there were no significant differences between the two groups while in both cases there was low heterogeneity (mITT: *I*^2^ = 26%; ITT: *I*^2^ = 5%). Certainty of evidence was low. Mortality was not presented by any of the trials in a “time-to-event” fashion. Therefore, as would be normally performed, a hazard ratio calculation could not be performed, and an odds ratio calculation had to be performed.

#### New multiple organ failure

New multiple organ failure was reported in all studies. There was a significant difference between the two groups in favor of the endoscopic group (mITT: OR [CI]: 0.31 [0.10, 0.98]; ITT: OR [CI]: 0.31 [0.10, 0.97]) with low heterogeneity (*I*^2^ = 0%) for both ITT and mITT. Certainty of evidence was moderate.

### Secondary outcomes

#### Perforation of visceral organ or enterocutaneous fistula

As not all trials reported perforations of visceral organs and enterocutaneous fistula separately, we pooled both reports. There were significantly more events in the surgical group in both mITT and ITT analysis (mITT: OR [CI]: 0.30 [0.10, 0.90]; ITT: OR [CI]: 0.31 [0.10, 0.93]) and heterogeneity was low (*I*^2^ = 0%). Certainty of evidence was low.

#### Pancreatic fistula

All trials reported occurrence of pancreatic fistula. One study only provided the number of pancreatic fistulae for patients that had not died to the 6-month follow up. After meta-analysis there were significantly more incidences of pancreatic fistula in the surgical group (mITT: OR [CI]: 0.08 [0.02, 0.25]; ITT: OR [CI]: 0.09 [0.03, 0.28]). Certainty of evidence was low.

#### Length of postintervention hospital stay

All studies reported length of hospital stay. However, the definitions varied. The PENGUIN-trial only reported the data of patients that did not die, while the TENSION-trial reported days in hospital after randomization and the MISER-trial reported days in hospital after the index procedure. Data were not available in order to perform an ITT analysis. The pooled available data showed significant results in favor of the endoscopic group (MD [CI]: − 7.86 [− 14.49, − 1.22]). Heterogeneity was low (*I*^2^ = 0%). However, this analysis does not represent the ITT analysis. Patients that recovered after drainage likely had a shorter hospital stay than the average and might have reduced the benefit that endoscopy seems to offer. The certainty of evidence was low due to the missing patient data.

#### Combined composite endpoint of all studies

All studies presented a composite score of patients that experienced either major complications or death. However, the interpretation of major complications differed between the studies as the PENGUIN-trial and the MISER-trial considered multiple organ failure and not single organ failure to be a major complication, whereas the TENSION-trial considered single organ failure to be a major complication. The composites were pooled, nonetheless. In both the mITT analysis and the ITT analysis there were no significant differences between the groups (mITT: OR [CI] 0.28 [0.06, 1.30]; ITT 0.36 [0.10, 1.27]) and heterogeneity was high in both analyses (mITT: *I*^2^ = 75%; ITT: *I*^2^ = 67%) which might have resulted from the differing definitions of the composites. The certainty of evidence was very low.

#### Bleeding requiring intervention

All studies reported bleeding that required intervention. There were no significant differences between the groups in both the mITT and ITT analysis (mITT: OR [CI] 0.57 [0.09, 3.74]; ITT: OR [CI] 0.60 [0.10, 3.59]) and heterogeneity was moderate (mITT: *I*^2^ = 44%; ITT = 41%). The certainty of evidence was moderate.

#### Incisional hernia

Incisional hernia was reported by two trials. Both mITT and the ITT analysis showed no significant differences (mITT: OR [CI] 0.23 [0.02, 2.11]; ITT: OR [CI] 0.24 [0.03, 2.18]) and heterogeneity was low (mITT: *I*^2^ = 0%; ITT: *I*^2^ = 0%). The certainty of evidence was moderate.

#### Exocrine insufficiency

All studies reported resulting exocrine insufficiency. There were differing definitions. There were no significant differences in both mITT and ITT analysis (mITT: OR [CI]: 0.74 [0.18, 3.03]; ITT: OR [CI]: 1.04 [0.31, 3.51]). Heterogeneity was moderate (mITT: *I*^2^ = 45%; ITT: *I*^2^ = 38%). The certainty of evidence was moderate.

#### Endocrine insufficiency

All studies reported resulting endocrine insufficiency. There were no significant differences in mITT analysis (mITT: OR [CI]: 0.80 [0.38, 1.68]). Heterogeneity was low (mITT: *I*^2^ = 0%). The certainty of evidence was moderate.

#### Length of ICU stay

All studies reported some kind of ICU stay; however, the definitions varied. The PENGUIN-trial reported total new ICU-admissions (Endoscopy: *n* = 1; Surgery: *n* = 5). The TENSION-trial reported the days spent in the ICU 6 months after randomization for patients not present in the ICU 24 h before randomization. The MISER-trial reported the days spent in ICU from the index procedure to discharge. We pooled the data provided by the TENSION-trial and the MISER-trial and there was no significant difference (MD [CI]: − 3.76 [− 8.33, 0.80]). Heterogeneity was low (*I*^2^ = 0%). However, this was not an ITT analysis and therefore might not represent the true outcome as the patients that recovered after drainage likely had a shorter ICU stay than average. The certainty of evidence was low due to the missing patient data.

#### Total costs

Cost analysis was performed in both the TENSION and the MISER-trial. Overall mean cost per patient was lower for the endoscopic treatment arm in both trials (TENSION: Endoscopy: 60 228 €; Surgery: 73 883 €; MISER: Endoscopy: 75 829 $; Surgery: 117 491 $). Due to the differences in health care systems and cost calculations, meta-analysis was not performed.

The visual representation of the ITT meta-analysis is presented in Table 3. For the visual representation of the mITT analysis please see Supplementary material.

**Table 3:**
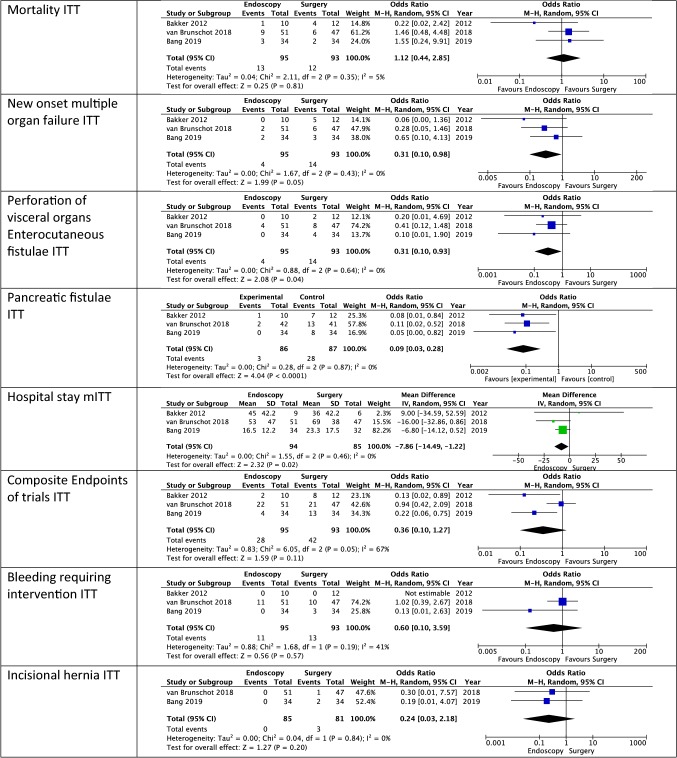

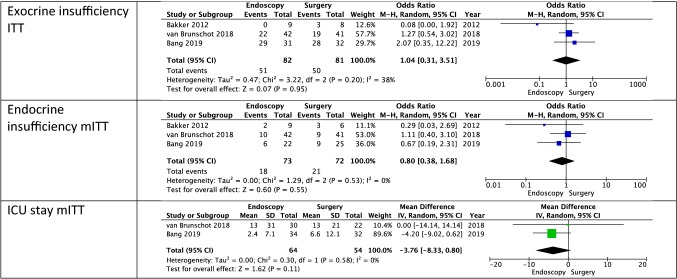
Meta-analysis of all outcomes (*ITT* intention to treat, *mITT* modified intention to treat)

The certainties of evidence of the outcomes that were considered most important are presented in Table 4. Certainty of evidence for all outcomes is presented in the supplementary material.

**Table 4:**
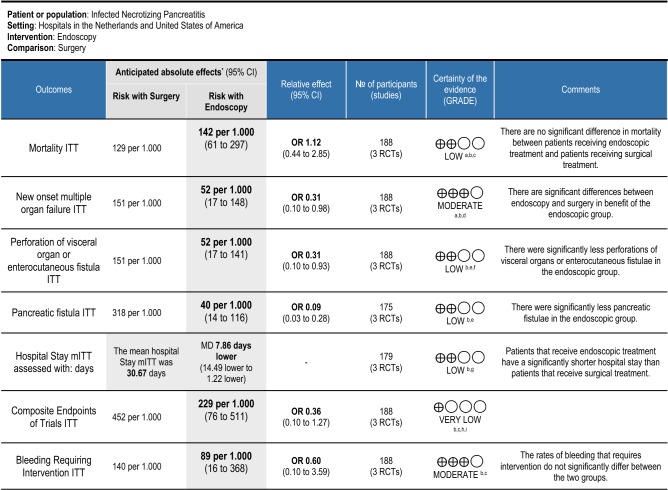
Summary of findings: endoscopy compared to surgery for infected necrotizing pancreatitis

GRADE Working Group grades of evidence: *High certainty* We are very confident that the true effect lies close to that of the estimate of the effect. *Moderate certainty* We are moderately confident in the effect estimate: The true effect is likely to be close to the estimate of the effect, but there is a possibility that it is substantially different. *Low certainty* Our confidence in the effect estimate is limited: The true effect may be substantially different from the estimate of the effect. *Very low certainty* We have very little confidence in the effect estimate: The true effect is likely to be substantially different from the estimate of effect

*CI* confidence interval, *OR* odds ratio, *MD* mean difference

^*^The risk in the intervention group (and its 95% confidence interval) is based on the assumed risk in the comparison group and the relative effect of the intervention (and its 95% CI)

^a^Objective Outcome, not at risk of bias

^b^Optimal Information size not reached

^c^Confidence intervals include significant benefit and significant harm

^d^Outcome well defined and at low risk of bias

^e^High risk of bias due to possible underestimation of frequency of fistulae due to lower rate of percutaneous drainage and resulting lower measurement of fistulae in endoscopic group

^f^No upgrading due to large effect due to possible confounding due to overdiagnosing in endoscopic group

^g^Not ITT analysis. Patient data missing (endoscopy: *n* = 1; surgery: *n* = 5)

^h^Possibly high risk of bias due to selection of reported results

^i^High Heterogeneity with *P* < 0.05

## Discussion

The present systematic review and meta-analysis compares the outcomes of endoscopy to surgery for INP based on the results of RCTs only. Since 2012, three trials comparing the two different approaches have been published. Both ITT and mITT analysis showed statistically significant differences in new onset multiple organ failure in favor of the endoscopic group. Furthermore, there were statistically significant differences in both ITT- and mITT analysis in favor of the endoscopic group for a composite of perforations of visceral organs and enterocutaneous fistulas, as well as for length of hospital stay and for pancreatic fistulas. No other significant differences were observed. Overall certainty of evidence was moderate.

### Key findings

While the meta-analysis did not show any significant differences in mortality between the two groups, this might possibly be due to the small number of studies that have explored the differences between these two approaches. The total number of patients added up to 190. Given this low sample size, differences in mortality would have to be very pronounced to be statistically significant. In order to make adequate assumptions concerning mortality, further trials are therefore needed.

The differences regarding higher rates of new onset multiple organ failure are particularly important as it has been shown that patients with INP with new onset single organ failure have a significantly higher mortality than patients without new organ failure [40]. It can be considered highly likely that this is the case with multiple organ failure as well. The underlying reason for this possibly greater rate of organ failure in patients with surgical treatment might be the higher inflammatory stress response that the surgical treatment causes in patients. This additional inflammatory response could aggravate already existing organ failure or induce new organ failure in already severely ill patients [41]. In addition, there were significantly more incidences of perforations of visceral organs or enterocutaneous fistulae in the surgical group and the hospital stay was shorter in the endoscopic group.

Overall, the results of this systematic review and meta-analysis have to be interpreted with caution. While endoscopic drainage and necrosectomy are relatively common procedures that have already been adopted by many physicians worldwide, the evidence remains scarce with less than 200 randomized patients due to the typical setting with often critically ill patients making inclusion into randomized trials a difficult venture. Nonetheless, this makes pooling the available data even more important as the single studies are small and taken alone may not adequately represent the underlying effects.

While the included studies selected patients in a similar way, there were differences between the studies. Patients in the MISER-trial might have generally been more ill as they showed higher ASA-status and a higher APACHE score in both the endoscopic and the surgery group when compared to the other two studies. In addition to this, the surgical interventions showed differences between the studies. While the PENGUIN-trial most often performed a step-up procedure with initial drainage and subsequent video-assisted retroperitoneal debridement (VARD), four procedures were performed in an open setting which is generally speaking seen as more physically demanding for the patients [2, 5]. The MISER-trial surgical group included two different procedures, one a laparoscopic transperitoneal procedure and the other a step-up procedure with initial percutaneous drainage and subsequent VARD. The TENSION-trial conducted a strict step-up approach in the surgical group with initial percutaneous drainage of all patients and if necessary subsequent VARD.

Nonetheless, the majority of patients underwent some sort of drainage with subsequent necrosectomy if required in the surgical group. Furthermore, by reincluding four patients initially excluded in two studies as they showed full recovery after drainage, the surgical interventions were more homogenous. By doing this we were able to perform an ITT analysis with relatively low overall meta-analysis heterogeneity. This might implicate that the actual underlying effect is based on general differences between the endoscopic and surgical approach.

In addition to the intertrial differences in the surgical procedures, there were differences concerning drainage techniques, placement of nasocystic catheters, and irrigation of the cyst in the endoscopic procedures between the different trials. In the MISER-trial, patients could be treated with a multi-gateway technique, and furthermore, the interventionists used 2.7 Fr plastic or metal stents whereas in the TENSION-trial only plastic stents were used. Interestingly, the same study group that performed the MISER-trial performed a trial comparing lumen-apposing metal stents and plastic stents for walled-off necrosis and they could not show any significant differences concerning treatment success or clinical adverse events [42]. However, the group with lumen-apposing metal stents showed a significantly higher number of stent-related adverse events. Therefore, these differences in stent usage might well add heterogeneity to the trials. Up to date, there is insufficient evidence to suggest which of these procedures is superior to the other but multicenter trials evaluating and comparing different procedures are ongoing [43–45].

One likely reason for the statistically significant difference of the main outcome of the MISER-trial, which was a composite outcome, was the inclusion of pancreatic fistulae into their composite. This is one of the main differences to the TENSION and the PENGUIN trials which both did not include pancreatic fistulae into the composite and has been noted in comments on the trial [46]. Even though pancreatic fistulae are a clinically important symptom, the inclusion into the composite outcome is likely what made the difference between statistical significance and insignificance, therefore introducing an important factor for bias into the primary outcome of the trial. Due to this, the risk of bias for the composite outcome was rated to be high and should be taken into account when interpreting this data.

One of the main developments of the last decade has been the introduction of drainage as a primary treatment option for patients with INP. While it will not suffice as the sole treatment for all patients, this step has shown the ability to spare a subgroup of patients the necessity of undergoing any kind of necrosectomy, be it surgical or endoscopic [47]. Further scrutinization of the different drainage procedures and evaluating potential benefits of one drainage procedure over others for different situations and patients is important. Furthermore, identifying patients for which the single step drainage is a viable option and differentiating these from patients that require intensified treatment in form of necrosectomy are important questions that will require further research. The future will likely show advantages for individualized treatment that combines the available treatment options to their advantage. Depending on localization and accessibility of INP in individual patients the optimal initial drainage, and if necessary, subsequent necrosectomy will have to be determined.

#### Strengths

We performed an extensive literature search and followed the recommendations of the Cochrane Handbook of Systematic Reviews and Interventions, the PRISMA guidelines and the AMSTAR-2 criteria. To our knowledge, this is the first review to include all RCTs scrutinizing this topic that have been published to date. By including novel methods of risk of bias assessment developed by the Cochrane group, we were able to more adequately identify risks of bias to the individual outcomes. By including patients of two trials that were excluded due to resolution of symptoms after drainage the interventions were more homogenous and comparable.

#### Limitations

Even though we included all available RCTs on this topic, evidence remains scarce with a total of under 200 patients having been randomized. The overall certainty remained moderate mainly due to this factor. While surgical interventions and certain technical aspects of the endoscopic procedure differed, after re-inclusion of patients excluded in two trials the interventions were deemed more homogenous.

#### Implications for further research

The quality of evidence was moderate for most outcomes. This indicates that further research might still impact the results. Further trials assessing the possibilities of endoscopic treatment compared to surgical treatment are warranted. In addition, the timing of the intervention has not yet been sufficiently examined. While the guidelines currently call for surgery to be delayed until the necrosis has become walled off, this practice dates back to the times of open surgery [48], a procedure that adds higher inflammatory response than minimally invasive treatment options. Trials assessing this question are underway and results are keenly awaited (ISRCTN33682933) [49]. Furthermore, the different combinations of percutaneous, endoscopic and minimally invasive procedures according to localization of necrotic lesions and fluid collections and resulting suitability for each treatment modality in the individual patient seem to logically warrant further research. Lastly, seeing as the endoscopic procedure presents a relatively novel procedure, many technical questions such as superiority of different stents as well as cystic drainage and irrigation remain to be examined in high quality randomized trials.

#### Implications for clinical practice

While the evidence still remains moderate, the results of this systematic review and meta-analysis point towards possible advantages of endoscopic treatment. However, as is with all new procedures, physicians adopting this technique should anticipate a learning curve and will have to cross this period taking adequate measures to ensure patient safety.

Furthermore, infected necrotizing pancreatitis will remain an illness that mandates individual patient evaluation in an obligatory multidisciplinary setting involving gastroenterologists, intensive care physicians, interventional radiologists and surgeons in order to adequately evaluate patient suitability for the different available interventions and treatment options.

Multidisciplinary treatment should combine the available options to their advantage. Depending on localization and accessibility of INP in individual patients the optimal initial drainage (percutaneous or endoscopic), and if necessary, subsequent necrosectomy (endoscopic or surgical) will be determined.

## Electronic supplementary material

Below is the link to the electronic supplementary material.

Electronic supplementary material 1 (DOCX 17 kb)

Electronic supplementary material 2 (DOCX 2073 kb)

Electronic supplementary material 3 (DOCX 19 kb)

Electronic supplementary material 4 (DOCX 21 kb)

Electronic supplementary material 5 (DOCX 14 kb)
