# Supplementary material for: Endoscopic versus surgical treatment for infected necrotizing pancreatitis: a systematic review and meta-analysis of randomized controlled trials
Source: Surg Endosc. 2020 Feb 28;34(6):2429–44. doi: 10.1007/s00464-020-07469-9 (PMC7214487; doi:10.1007/s00464-020-07469-9)
Supplement: Supplementary file 1 — Electronic supplementary material 1 (DOCX 17 kb) [file 464_2020_7469_MOESM1_ESM.docx]

Table 1: Outcome assessment as presented by the trials

| Outcome | PENGUIN trial | TENSION trial | MISER trial |
| --- | --- | --- | --- |
| Mortality | Patient death | Patient death | Patient death |
| Organ failure | - Pulmonary Failure: PaO2 lower than 60 mm Hg despite fraction of inspired oxy- gen (FIO2) of 30% or need for mechanical ventilation - Circulatory Failure: Circulatory systolic blood pressure below 90 mm Hg de- spite adequate fluid resuscitation or need for inotropic cat- echolamine support - Renal Failure: Creatinine level more than 2.0 mg/dL after rehydration or new need for hemofiltration or hemodialysis | - Pulmonary: a PaO2 < 60 mmHg despite FiO2 30%, or the need for mechanical ventilation - Cardiovascular: a systolic blood pressure < 90 mmHg despite adequate fluid resuscitation or need for vasopressor support - Renal: a serum creatinine > 177 mmol/L after rehydration or need for hemofiltration or hemodialysis (in case patients already suffered from renal insufficiency before this episode of AP [creatinine > 177 umol/L] this does not count as renal failure) | - Respiratory failure: PaO2<60 mm Hg on FiO230% or requiring ventilatory support  - Cardiovascular failure: Systolic blood pressure<90 mm Hg following sufficient intravascular resuscitation or administration of inotropes for pressure support  - Renal failure: Rise in serum creatinine by>3-fold or oliguria (urine output<0.3 mL/kg per h) or newly requiring hemofiltration/hemodialysis  - Gastrointestinal failure: Blood loss from the gastrointestinal tract>500 mL or ischemia/infarction of the gastrointestinal tract  - Hepatic failure: At least 3-fold elevation in AST/ALT with elevation in INR (>1.5) and encephalopathy  - Hematological failure: Disseminated intravascular coagulation defined as prolonged PT/PTT, platelet count<100,000/mL, schistocytes, and/or elevated fibrin degradation products  - Neurological failure: Glasgow coma scale<13 or encephalopathy  - Metabolic failure: Serum calcium<7.5 mg/dL or metabolic acidosis/alkalosis |
| New onset multiple organ failure | Failure of 2 or more organs at the same time | Organ failure occurring after randomisation and not present 24 hours before randomisation: Failure of 2 or more organ systems (respiratory, cardiovascular or renal) at the same moment | New-onset multiple organ failure: New-onset failure of >=2 organs within 48 hours of intervention |
| Composite of Perforating of visceral organ and enterocutaneous fistula | Enterocutaneous Fistula or Perforation of a Visceral Organ Requiring Intervention Secretion of fecal material from a percutaneous drain or drain- age canal after removal of drains or from a surgical wound; secretion comes from either the small or large bowel and is con- firmed with imaging or during surgery and requires either surgi- cal, radiological, or endoscopic intervention | Perforation of viasceral organ: Requiring surgical, radiologic, or endoscopic intervention  Enterocutaneous fistula: Secretion of fecal material from a percutaneous drain or drainage canal after removal of drains or from a surgical wound, either from small or large bowel; confirmed by imaging or during surgery | Perforation of a visceral organ requiring interventional procedure  Formation of a fistula between the small bowel or colon and skin as indicated by feculent output from a percutaneous catheter or surgical wound site, confirmed on imaging or surgery |
| Pancreatic fistula | Output via a percutaneous or nasocystic drain or drainage canal after removal of percutaneous drains or from a surgical wound of any measurable volume of fluid with an amylase content greater than 3 times the serum amylase activityb | Output, through a percutaneous drain or drainage canal after removal of drains from a surgical wound, or any measurable volume of fluid with an amylase content > 3 times the serum amylase level | Formation of a fistula between the pancreas and skin as indicated by amylase-rich fluid output from a percutaneous catheter or surgical wound site, persisting after3 months, and requiring specific treatment that include parenteral or enteral nutrition, antibiotics, somatostatin analogues and minimally invasive drainage |
| Hospital stay | Surviving patients only, from randomization onwards | 6 months after randomization | From index intervention to discharge |
| Composite endpoint | Death, new onset multiple organ failure, intra-abdominal bleeding requiring intervention, enterocutaneous fistula or perforation of visceral organ requiring intervention, pancreatic fistula | composite of major complications or death within 6 months after random­ isation. Major complications were defined as new­onset organ failure (ie, cardiovascular, pulmonary, or renal), bleeding requiring intervention, perforation of a visceral organ requiring intervention (except for the intentionally made perforation during endopscopic treatment), enterocutaneous fistula requiring intervention, and incisional hernia (including burst abdomen). | composite of major complications comprising new-onset multiple organ failure or systemic dysfunction, enteral or pancreatic-cutaneous fistula, intra-abdominal bleeding, visceral perforation, or death during admission and until 6 months after discharge. |
| Bleeding requiring intervention | Intra-abdominal Bleeding Requiring Surgical, radiological, or endoscopic intervention | Requiring surgical, radiologic, or endoscopic intervention | Bleeding within the intraabdominal cavity or gastrointestinal tract requiring interventional procedure |
| Incisional hernia | Not assessed | Full-thickness discontinuity in abdominal wall and bulging of abdominal contents, with or without obstruction | Herniation of intra-abdominal structures through an area of weakness in theabdominal fascia, requiring surgical intervention or persisting at 6-month follow-up |
| Endocrine insufficiency | New-Onset Diabetes: The need for insulin or oral antidiabetic drugs to treat diabetes—which was not present before pancreatitis—6 months after discharge | Insulin or oral antidiabetic drugs required 6 months after randomisation; this requirement was not present before onset of pancreatitis | New onset elevation in fasting plasma glucose >=126 mg/dL, 2-hour plasma glucose >=200 mg/dL after an oral glucose tolerance test or HbA1c>=6.5% |
| Exocrine insufficiency | Use of Pancreatic Enzymes: The use of oral pancreatic enzyme supplementation to treat clinical symptoms of steatorrhea—which was not present before on- set of pancreatitis—6 months after discharge | Oral pancreatic-enzyme supplementation required to treat clinical symptoms of steatorrhea 6 months after randomisation; this requirement was not present before onset of pancreatitis | Fecal elastase level<200mg/g in patients not previously taking pancreatic enzyme supplements |
| ICU stay | Not assessed | 6 months after randomization and only for patients not in ICU at randomization | From index intervention to discharge |
