# Supplementary material for: Endoscopic versus surgical treatment for infected necrotizing pancreatitis: a systematic review and meta-analysis of randomized controlled trials
Source: Surg Endosc. 2020 Feb 28;34(6):2429–44. doi: 10.1007/s00464-020-07469-9 (PMC7214487; doi:10.1007/s00464-020-07469-9)
Supplement: Supplementary file 2 — Electronic supplementary material 2 (DOCX 2073 kb) [file 464_2020_7469_MOESM2_ESM.docx]

Table 2: Full ITT and mITT analysis

| Mortality mITT |  |
| --- | --- |
| Mortality ITT |  |
| New onset multiple organ failure mITT |  |
| New onset multiple organ failure ITT |  |
| Enterocutaneous fistulae and perforation of visceral organs mITT |  |
| Enterocutaneous fistulae and perforation of visceral organs ITT |  |
| Pancreatic fistulae mITT |  |
| Pancreatic fistulae ITT |  |
| Hospital stay mITT |  |
| ICU stay mITT |  |
| Composite Endpoints of trials mITT |  |
| Composite Endpoints of trials ITT |  |
| Bleeding requiring intervention mITT |  |
| Bleeding requiring intervention ITT |  |
| Incisional hernia mITT |  |
| Incisional hernia ITT |  |
| Exocrine insufficiency mITT |  |
| Exocrine insufficiency ITT |  |
| Endocrine insufficiency mITT |  |
