# Supplementary material for: Endoscopic versus surgical treatment for infected necrotizing pancreatitis: a systematic review and meta-analysis of randomized controlled trials
Source: Surg Endosc. 2020 Feb 28;34(6):2429–44. doi: 10.1007/s00464-020-07469-9 (PMC7214487; doi:10.1007/s00464-020-07469-9)
Supplement: Supplementary file 3 — Electronic supplementary material 3 (DOCX 19 kb) [file 464_2020_7469_MOESM3_ESM.docx]

| **Summary of findings:** | | | | | | |
| --- | --- | --- | --- | --- | --- | --- |
| **Endoscopy compared to Surgery for Infected Necrotizing Pancreatitis** | | | | | | |
| **Patient or population**: Infected Necrotizing Pancreatitis  **Setting**: Hospitals in the Netherlands and United States of America  **Intervention**: Endoscopy  **Comparison**: Surgery | | | | | | |
| Outcomes | **Anticipated absolute effects^*^** (95% CI) | | Relative effect (95% CI) | № of participants  (studies) | Certainty of the evidence (GRADE) | Comments |
|  | **Risk with Surgery** | **Risk with Endoscopy** |  |  |  |  |
| Mortality ITT | 129 per 1.000 | **142 per 1.000** (61 to 297) | **OR 1.12** (0.44 to 2.85) | 188 (3 RCTs) | ⨁⨁◯◯ LOW ^a,b,c^ | There are no significant difference in mortality between patients receiving endoscopic treatment and patients receiving surgical treatment. |
| New onset multiple organ failure ITT | 151 per 1.000 | **52 per 1.000** (17 to 148) | **OR 0.31** (0.10 to 0.98) | 188 (3 RCTs) | ⨁⨁⨁◯ MODERATE ^a,b,d^ | There are significant differences between endoscopy and surgery in benefit of the endoscopic group. |
| Perforation of visceral organ or enterocutaneous fistula ITT | 151 per 1.000 | **52 per 1.000** (17 to 141) | **OR 0.31** (0.10 to 0.93) | 188 (3 RCTs) | ⨁⨁◯◯ LOW ^b,e,f^ | There were significantly less perforations of visceral organs or enterocutaneous fistulae in the endoscopic group. |
| Pancreatic fistula ITT | 318 per 1.000 | **40 per 1.000** (14 to 116) | **OR 0.09** (0.03 to 0.28) | 175 (3 RCTs) | ⨁⨁◯◯ LOW ^b,e^ | There were significantly less pancreatic fistulae in the endoscopic group. |
| Hospital Stay mITT assessed with: days | The mean hospital Stay mITT was **30.67** days | MD **7.86 days lower** (14.49 lower to 1.22 lower) | - | 179 (3 RCTs) | ⨁⨁◯◯ LOW ^b,g^ | Patients that receive endoscopic treatment have a significantly shorter hospital stay than patients that receive surgical treatment. |
| Composite Endpoints of Trials ITT | 452 per 1.000 | **229 per 1.000** (76 to 511) | **OR 0.36** (0.10 to 1.27) | 188 (3 RCTs) | ⨁◯◯◯ VERY LOW ^b,c,h,i^ |  |
| Bleeding Requiring Intervention ITT | 140 per 1.000 | **89 per 1.000** (16 to 368) | **OR 0.60** (0.10 to 3.59) | 188 (3 RCTs) | ⨁⨁⨁◯ MODERATE ^b,c^ | The rates of bleeding that requires intervention do not significantly differ between the two groups. |
| Incisional Hernia ITT | 37 per 1.000 | **9 per 1.000** (1 to 77) | **OR 0.24** (0.03 to 2.18) | 166 (2 RCTs) | ⨁⨁⨁◯ MODERATE ^b,c^ |  |
| Exocrine Insufficiency ITT | 543 per 1.000 | **553 per 1.000** (269 to 807) | **OR 1.04** (0.31 to 3.51) | 163 (3 RCTs) | ⨁⨁⨁◯ MODERATE ^b,c^ |  |
| Endocrine Insufficiency mITT | 292 per 1.000 | **248 per 1.000** (135 to 409) | **OR 0.80** (0.38 to 1.68) | 145 (3 RCTs) | ⨁⨁⨁◯ MODERATE ^b,c^ |  |
| ICU Stay mITT | The mean ICU Stay mITT was **0** | MD **3.76 lower** (8.33 lower to 0.8 higher) | - | 118 (2 RCTs) | ⨁⨁◯◯ LOW ^b,g^ | There is no difference in ICU stay in patients that receive endoscopic treatment and patients that receive surgical treatment. |
| ***The risk in the intervention group** (and its 95% confidence interval) is based on the assumed risk in the comparison group and the **relative effect** of the intervention (and its 95% CI).   **CI:** Confidence interval; **OR:** Odds ratio; **MD:** Mean difference | | | | | | |
| **GRADE Working Group grades of evidence** **High certainty:** We are very confident that the true effect lies close to that of the estimate of the effect **Moderate certainty:** We are moderately confident in the effect estimate: The true effect is likely to be close to the estimate of the effect, but there is a possibility that it is substantially different **Low certainty:** Our confidence in the effect estimate is limited: The true effect may be substantially different from the estimate of the effect **Very low certainty:** We have very little confidence in the effect estimate: The true effect is likely to be substantially different from the estimate of effect | | | | | | |

#### Explanations

a. Objective Outcome, not at risk of bias

b. Optimal Information size not reached

c. Confidence intervals include significant benefit and significant harm

d. Outcome well defined and at low risk of bias

e. High risk of bias due to possible underestimation of frequency of fistulae due to lower rate of percutaneous drainage and resulting lower measurement of fistulae in endoscopic group.

f. No upgrading due to large effect due to possible confounding due to overdiagnosing in endoscopic group.

g. Not ITT analysis. Patient data missing (Endoscopy: n = 1; Surgery: n = 5)

h. Possibly high risk of bias due to Selection of reported results

i. High Heterogeneity with P < 0.05
