# Supplementary material for: Endoscopic versus surgical treatment for infected necrotizing pancreatitis: a systematic review and meta-analysis of randomized controlled trials
Source: Surg Endosc. 2020 Feb 28;34(6):2429–44. doi: 10.1007/s00464-020-07469-9 (PMC7214487; doi:10.1007/s00464-020-07469-9)
Supplement: Supplementary file 4 — Electronic supplementary material 4 (DOCX 21 kb) [file 464_2020_7469_MOESM4_ESM.docx]

**Author(s)**: Haney et al

**Question**: Endoscopy compared to Surgery for Infected Necrotizing Pancreatitis

**Setting**: Hospitals in the Netherlands and United States of America

**Bibliography**:

| **Certainty assessment** | | | | | | | **№ of patients** | | **Effect** | | **Certainty** | **Importance** |
| --- | --- | --- | --- | --- | --- | --- | --- | --- | --- | --- | --- | --- |
| **№ of studies** | **Study design** | **Risk of bias** | **Inconsistency** | **Indirectness** | **Imprecision** | **Other considerations** | **Endoscopy** | **Surgery** | **Relative (95% CI)** | **Absolute (95% CI)** |  |  |
| **Mortality ITT** | | | | | | | | | | | | |
| 3 | randomised trials | not serious ^a^ | not serious | not serious | very serious ^b,c^ | none | 13/95 (13.7%) | 12/93 (12.9%) | **OR 1.12** (0.44 to 2.85) | **13 more per 1.000** (from 68 fewer to 168 more) | ⨁⨁◯◯ LOW | CRITICAL |
| **New onset multiple organ failure ITT** | | | | | | | | | | | | |
| 3 | randomised trials | not serious ^a,d^ | not serious | not serious | serious ^b^ | none | 4/95 (4.2%) | 14/93 (15.1%) | **OR 0.31** (0.10 to 0.98) | **98 fewer per 1.000** (from 133 fewer to 3 fewer) | ⨁⨁⨁◯ MODERATE | CRITICAL |
| **Perforation of visceral organ or enterocutaneous fistula ITT** | | | | | | | | | | | | |
| 3 | randomised trials | serious ^e^ | not serious | not serious | serious ^b^ | none ^f^ | 4/95 (4.2%) | 14/93 (15.1%) | **OR 0.31** (0.10 to 0.93) | **98 fewer per 1.000** (from 133 fewer to 9 fewer) | ⨁⨁◯◯ LOW | CRITICAL |
| **Pancreatic fistula ITT** | | | | | | | | | | | | |
| 3 | randomised trials | serious ^e^ | not serious | not serious | serious ^b^ | none | 3/87 (3.4%) | 28/88 (31.8%) | **OR 0.09** (0.03 to 0.28) | **278 fewer per 1.000** (from 304 fewer to 203 fewer) | ⨁⨁◯◯ LOW | IMPORTANT |
| **Hospital Stay mITT (assessed with: days)** | | | | | | | | | | | | |
| 3 | randomised trials | serious ^g^ | not serious | not serious | serious ^b^ | none | 94 | 85 | - | MD **7.86 days lower** (14.49 lower to 1.22 lower) | ⨁⨁◯◯ LOW | IMPORTANT |
| **Composite Endpoints of Trials ITT** | | | | | | | | | | | | |
| 3 | randomised trials | serious ^h^ | serious ^i^ | not serious | serious ^b,c^ | none | 28/95 (29.5%) | 42/93 (45.2%) | **OR 0.36** (0.10 to 1.27) | **223 fewer per 1.000** (from 376 fewer to 60 more) | ⨁◯◯◯ VERY LOW | NOT IMPORTANT |
| **Bleeding Requiring Intervention ITT** | | | | | | | | | | | | |
| 3 | randomised trials | not serious | not serious | not serious | serious ^b,c^ | none | 11/95 (11.6%) | 13/93 (14.0%) | **OR 0.60** (0.10 to 3.59) | **51 fewer per 1.000** (from 124 fewer to 229 more) | ⨁⨁⨁◯ MODERATE | IMPORTANT |
| **Incisional Hernia ITT** | | | | | | | | | | | | |
| 2 | randomised trials | not serious | not serious | not serious | serious ^b,c^ | none | 0/85 (0.0%) | 3/81 (3.7%) | **OR 0.24** (0.03 to 2.18) | **28 fewer per 1.000** (from 36 fewer to 40 more) | ⨁⨁⨁◯ MODERATE | NOT IMPORTANT |
| **Exocrine Insufficiency ITT** | | | | | | | | | | | | |
| 3 | randomised trials | not serious | not serious | not serious | serious ^b,c^ | none | 45/82 (54.9%) | 44/81 (54.3%) | **OR 1.04** (0.31 to 3.51) | **10 more per 1.000** (from 274 fewer to 264 more) | ⨁⨁⨁◯ MODERATE | IMPORTANT |
| **Endocrine Insufficiency mITT** | | | | | | | | | | | | |
| 3 | randomised trials | not serious | not serious | not serious | serious ^b,c^ | none | 18/73 (24.7%) | 21/72 (29.2%) | **OR 0.80** (0.38 to 1.68) | **44 fewer per 1.000** (from 156 fewer to 117 more) | ⨁⨁⨁◯ MODERATE | IMPORTANT |
| **ICU Stay mITT** | | | | | | | | | | | | |
| 2 | randomised trials | serious ^g^ | not serious | not serious | serious ^b^ | none | 64 | 54 | - | MD **3.76 lower** (8.33 lower to 0.8 higher) | ⨁⨁◯◯ LOW | IMPORTANT |

**CI:** Confidence interval; **OR:** Odds ratio; **MD:** Mean difference

#### Explanations

a. Objective Outcome, not at risk of bias

b. Optimal Information size not reached

c. Confidence intervals include significant benefit and significant harm

d. Outcome well defined and at low risk of bias

e. High risk of bias due to possible underestimation of frequency of fistulae due to lower rate of percutaneous drainage and resulting lower measurement of fistulae in endoscopic group.

f. No upgrading due to large effect due to possible confounding due to overdiagnosing in endoscopic group.

g. Not ITT analysis. Patient data missing (Endoscopy: n = 1; Surgery: n = 5)

h. Possibly high risk of bias due to Selection of reported results

i. High Heterogeneity with P < 0.05
