# Supplementary material for: Endoscopic versus surgical treatment for infected necrotizing pancreatitis: a systematic review and meta-analysis of randomized controlled trials
Source: Surg Endosc. 2020 Feb 28;34(6):2429–44. doi: 10.1007/s00464-020-07469-9 (PMC7214487; doi:10.1007/s00464-020-07469-9)
Supplement: Supplementary file 5 — Electronic supplementary material 5 (DOCX 14 kb) [file 464_2020_7469_MOESM5_ESM.docx]

Search syntax:

Pubmed:

(((necrot*[tiab] AND (pancreatitis[tiab] OR pancreatic[tiab] OR pancreas[tiab])) OR "Pancreatitis, Acute Necrotizing"[Mesh]

AND

(endoscop*[tiab] OR surgery[tiab] OR surgeries[tiab] OR surgical[tiab] OR operation*[tiab] OR therap*[tiab] OR “minimally invasive”[tiab] OR “endoscopic transgastric approach”[tiab] OR ETA[tiab] OR necrosectom*[tiab] OR transgastric[tiab] OR transluminal[tiab]))

OR "Pancreatitis, Acute Necrotizing/surgery"[Mesh] OR "Pancreatitis, Acute Necrotizing/therapy"[Mesh])
AND

random*[tiab] OR RCT*[tiab] OR “Randomized Controlled Trial”[pt] OR "Randomized Controlled Trials as Topic"[Mesh] OR "Controlled Clinical Trials as Topic"[Mesh]

Web of Science:

TS = (necrot* NEAR (pancreatitis OR pancreatic OR pancreas))

AND

TS = (endoscop* OR surgery OR surgeries OR surgical OR operation* OR therap* OR “minimally invasive” OR “endoscopic transgastric approach” OR ETA OR necrosectom* OR transgastric OR transluminal)

AND

TS = (random* OR RCT*)

CENTRAL:

(necrot* NEAR (pancreatitis OR pancreatic OR pancreas)):ti,ab,kw

OR "Pancreatitis, Acute Necrotizing"[Mesh]

AND

(endoscop* OR surgery OR surgeries OR surgical OR operation* OR therap* OR “minimally invasive” OR “endoscopic transgastric approach” OR ETA OR necrosectom* OR transgastric OR transluminal):ti,ab,kw

OR "Pancreatitis, Acute Necrotizing/surgery"[Mesh] OR "Pancreatitis, Acute Necrotizing/therapy"[Mesh])
